# The Impact of Morning Meditation and Sleep Quality on Affective and Health Outcomes in Healthcare Workers

**DOI:** 10.3390/ijerph22040592

**Published:** 2025-04-09

**Authors:** Ana Junça-Silva, Marisa Kulyk, António Caetano

**Affiliations:** 1 Business Research Unit (BRU-UNIDE-IUL), Instituto Universitário de Lisboa (ISCTE—IUL), 1649-026 Lisbon, Portugal; marisa.kulyk@ipt.pt (M.K.); antonio.caetano@iscte-iul.pt (A.C.); 2 APPSYCI—Applied Psychology Research Center Capabilities & Inclusion_APPsyCI, ISPA, 1149-041 Lisboa, Portugal

**Keywords:** meditation practices, sleep quality, health, mental health, vitality, positive affect, micro-breaks

## Abstract

Background: Health is a critical factor influencing key workplace outcomes, including job attitudes, behaviors, and performance. This study investigated the role of daily micro-breaks, specifically morning meditation practices, and positive affective experiences (i.e., positive affect) at work in predicting health-related outcomes, namely vitality and mental health. Using a non-experimental design, this study tested a moderated mediation model in which sleep quality moderates the relationship between morning meditation and positive affect, which, in turn, predicts end-of-the-day health-related outcomes. Methodology: Data were collected twice a day from 44 healthcare employees over five consecutive workdays using a daily survey approach. Key Results: Multilevel modeling analyses revealed that morning meditation was significantly associated with increased positive affect and improved health indicators at the end of the workday. Moreover, sleep quality moderated the relationship between morning meditation and positive affect, such that the conditional indirect effect of meditation on end-of-day mental health and vitality via positive affect was significant when sleep quality from the preceding night was poor. Conclusions: These findings underscore the interactive effect of prior-night sleep quality and morning meditation on affective and health-related outcomes by the end of the day. By identifying sleep quality as a key boundary condition, we contribute to a more nuanced understanding of when meditation is most beneficial. Our findings have significant implications for both research and practice, particularly in high-stress environments such as healthcare, where optimizing employee well-being is crucial for both individual and organizational performance.

## 1. Introduction

Meditation is widely recognized as an effective coping strategy for employees navigating challenging and demanding workdays [1]. It serves as an umbrella term encompassing a diverse range of practices, all sharing a fundamental goal: enhancing attentional control to improve awareness, concentration, nonjudgmental attitudes, and acceptance [2]. Additionally, meditation fosters deep relaxation, mental clarity, and inner peace [3]. At its core, meditation involves the deliberate cultivation of heightened awareness and self-regulation, enabling individuals to exert greater control over their thoughts and emotions [4].

Empirical research has demonstrated that meditation induces significant neurological changes, reinforcing key brain regions involved in stress regulation and emotional processing [5]. Specifically, meditation increases activity in the prefrontal cortex, cingulate cortex, and hippocampus while concurrently reducing activity in the amygdala—an area critical for processing emotions, particularly fear and stress [6]. Notably, studies have shown that sustained meditation practice can lead to a reduction in both the size and reactivity of the amygdala, aligning with findings of decreased stress and anxiety levels [7,8]. This downregulation enhances emotional regulation, fostering greater stability, resilience, and psychological well-being [9]. Additionally, meditation appears to modulate activity within the orbitofrontal cortex and anterior cingulate cortex, regions implicated in pain perception and emotional regulation [10]. These neural adaptations likely contribute to the relaxation and overall improvements in mental and physical health associated with meditation [11].

Meditation also enhances functional connectivity among various brain regions. Functional magnetic resonance imaging (fMRI) studies have demonstrated increased connectivity between the prefrontal cortex and the default mode network (DMN), a network linked to self-referential thinking and mind-wandering [12]. In experienced meditators, DMN activity is significantly reduced during meditation, which has been associated with decreased rumination and mind-wandering—two cognitive patterns frequently linked to anxiety and depression [13,14]. Over time, regular meditation practice enables individuals to respond more positively to daily events, thereby enhancing their experience of positive affect—short-lived emotions such as enthusiasm [15,16]. For instance, Fredrickson et al. [17] found that mindfulness and compassion meditation significantly increased individuals’ daily pleasure, assessed as an aggregate of ten positive emotions.

The broaden-and-build theory of positive emotions [18] posits that experiencing positive emotions expands individuals’ momentary thought–action repertoires, facilitating the development of valuable personal resources, including physical (e.g., subjective vitality) and psychological (e.g., mental health) well-being. The ability to cultivate and sustain positive emotions is therefore regarded as a fundamental mechanism for human flourishing and performance [19]. Furthermore, from the perspective of resource conservation theory [20], expanding and developing these resources strengthens individuals’ ability to acquire and protect other critical resources, such as health, thereby contributing to long-term well-being.

Previous studies have demonstrated that short meditation sessions during the workday positively impact well-being, positive affect, and health [21]. However, research has yet to fully explore the role of meditation during micro-breaks and its influence on affective and health-related outcomes [22]. Furthermore, limited attention has been given to identifying the conditions that may either enhance or mitigate the beneficial effects of meditation, with most studies focusing on relatively stable characteristics such as personality traits [11].

Drawing on the broaden-and-build theory, we propose that brief moments of daily meditation help employees relax, making them more receptive to experiencing positive affect during the workday. This, in turn, generates energy (i.e., vitality) and promotes mental health by the end of the day. However, given the increasing prevalence of sleep difficulties among the working population [23], we also argue that sleep quality from the preceding night serves as a boundary condition in this relationship. Specifically, considering the well-documented role of sleep as a crucial recovery process for replenishing lost resources [24], we hypothesize that when individuals experience poor sleep quality, meditation becomes even more relevant in fostering positive affect and, consequently, improving health outcomes. This study thus aims to examine how and when meditation influences employees’ health at the end of the workday, with positive affect as a momentary mechanism and sleep quality as a dynamic moderating factor.

This study makes several theoretical contributions. First, it advances the literature on workplace meditation and health by demonstrating that meditation during micro-breaks can enhance affective experiences and health outcomes, thereby expanding current knowledge on how meditation can be effectively integrated into organizational settings. While prior research has primarily investigated meditation as a predictor of well-being and positive emotions, this study shifts the focus to the mechanisms and contingencies that shape the effectiveness of meditation during work-related micro-breaks. In doing so, we respond to Fendel et al.’s [25] call to move beyond well-being and calmness as primary outcomes of meditation and to consider health indicators as critical components of employees’ quality of work life.

Additionally, this study identifies conditions under which the benefits of meditation may be amplified. Specifically, we examine sleep quality as a boundary condition influencing the relationship between workplace meditation, positive affect, and health (i.e., vitality and mental well-being). This aligns with Hülsheger et al.’s [26] call for research exploring not only whether meditation is effective in workplace settings [27] but also for whom it is most beneficial. Addressing this gap, our findings suggest that a good night’s sleep strengthens the association between meditation during micro-breaks and improved affective and health outcomes. By highlighting the unique role of sleep in this dynamic process, our study deepens understanding of how meditation interacts with daily sleep patterns to shape employees’ well-being.

Furthermore, this study advances the literature on healthcare workers’ well-being by addressing the significant challenges they encounter in their demanding profession. Healthcare professionals often endure long shifts, high-pressure environments, and unpredictable situations that necessitate constant adaptability [28]. The inherent uncertainty of their work—ranging from rapidly evolving patient conditions to unforeseen emergencies—exacerbates stress and emotional strain [29]. These intense demands can lead to physical exhaustion, cognitive overload, and emotional fatigue, ultimately compromising both well-being and job performance [30]. Additionally, the psychological burdens associated with crisis situations not only affect healthcare workers’ mental health but may also jeopardize patient safety [31].

Given these challenges, understanding how and when meditation can serve as an effective coping tool is essential. As a structured practice that enhances self-regulation, emotional resilience, and stress management [32], meditation offers a promising strategy to mitigate the cognitive and emotional demands inherent in healthcare settings. By incorporating meditation into their daily routines, healthcare workers may develop greater psychological flexibility, allowing them to navigate workplace pressures more effectively while safeguarding both their well-being and the quality of patient care.

Moreover, recognizing sleep quality as a moderating factor informs the development of targeted interventions to optimize meditation practices for healthcare professionals. This study highlights that workplace meditation is most effective when paired with restorative sleep, providing valuable insights for designing evidence-based interventions tailored to the specific needs of healthcare workers. Organizations could implement structured meditation programs during shift breaks, integrate sleep hygiene training into wellness initiatives, and cultivate a workplace culture that prioritizes both mindfulness and adequate rest. By fostering these practices, healthcare institutions can better support their employees’ mental health and resilience, ultimately enhancing both workforce sustainability and patient outcomes.

## 2. Theoretical Framework

### 2.1. Meditation

The diversity of practices that meditation encompasses has made it difficult to create a comprehensive and inclusive definition of what it means [6]. For instance, Shapiro [33,34] defined meditation as a practice that encompasses a set of techniques whose goal is the development of the focus of attention. That is, it encompasses “self-regulation practices focused on training attention and awareness to make mental processes more controllable by the individual and, thus, promote well-being and general mental development and/or specific abilities, such as calmness, clarity, and concentration” [34] (p. 228).

Furthermore, meditation has been described as a practice with similar results to those obtained with some cognitive psychotherapy techniques, although using different resources and techniques [35,36]; both techniques are focused on the development of skills to help individuals effectively deal with automatic thoughts, leading thereby to a decrease in their repetitive thinking and to a cognitive re-orientation. Thus, meditation has also been designated as a mind–body technique [36], a behavioral technique [37], or a mental relaxation response [38,39] capable of producing a greater integration between the mind, the body and the external world [40,41], as it develops skills for individuals to react more favorably to daily hassles or stressful events.

Some of the most applied practices in the work context are those with origins in the Buddhist tradition, such as mindfulness or loving-kindness meditation. The first one is an open meditation practice that considers the perception of stimuli such as thoughts, emotions, and sensations [42] and is focused on the maintenance of a free observation without judgments or interpretations, such as Zen meditation and Vipassana meditation. It has been widely used in organizations, management, and entrepreneurship studies (e.g., [43,44]). The loving-kindness meditation aims to develop self-compassion [45], allowing individuals, instead of being self-critical, to be kinder to themselves and experience less fear or anxiety concerning different situations [46], including in the work context.

### 2.2. The Relationship Between Meditation and Affect

Although there are several meditation techniques, research has shown that all of them have two common characteristics—attention control [47] or mindfulness [48]—and lead to one common outcome—higher frequency of positive affect (e.g., [15]).

Affect is an umbrella term that includes emotions (both positive and negative), stress, arousal, and mood [19]. Emotions refer to affective experiences that can be intense and short-lived, focused on specific objects or events [49]. The experience of positive affect is important for resilience, flourishing, vitality, happiness, and mental health [50], including in the workplace.

Indeed, meditation practices can turn attention to the present moment without judgment, reaction, or interference [51] and due to these changes, they improve awareness (creating a broader focus on everything that comes or goes out of consciousness) and attention (directs the focus to a specific stimulus or experience; [52]). Hence, meditation not only directs awareness to what happens in the present but also regulates sustained attention to what happens [53], improving the individual’s ability to regulate emotions [54].

The modal model of emotion described by Valim et al. [55] helps to understand how meditation practices may positively influence individuals’ affective responses. Accordingly, emotions are created through four stages: (1) a situation (stimulus) that is (2) attended to (focus) and then (3) appraised (evaluation), which creates (4) an affective response (emotions, e.g., enthusiasm or sadness) [56]. Accordingly, affective responses are coordinated and regulated reactions and represent a flexible multisystem, including, for instance, changes to the autonomic nervous system, facial expressions, non-verbal behaviors, actions, and attitudes. When individuals are aware of what happens, these multisystem reactions are easily noticed, and as such, may influence them to change their focus of attention to reappraise the situation and thereby shape their affective responses. In other words, based on this model, the more the individual pays attention to what happens and focuses on it, the more s/he will understand it, improving the likelihood of evaluating it in a positive way, which, in turn, will lead him/her to experience positive affect. Thereby, meditation may improve awareness of what happens, and increase the sustained focus on specific stimuli (e.g., events) which may improve the individual’s ability to appraise them neutrally or positively, even if the stimulus is negative [17]. Appraising stimulus more neutrally may make the individual experience more positive affective reactions [57].

The positive relationship between meditation and positive affect is consensual, as meditating creates positivity that influences emotions [58], and helps in their effective management [33]. For instance, Baer [59] showed that compassion-focused meditation significantly predicted positive affect. In addition, the study by Förster et al. [60] with patients with rheumatoid arthritis demonstrated that meditation increased functioning within corticosteroid circuits, inhibited pain, and increased positive affect concerning active control (non-conscious breathing). Fredrickson et al. [15], in a 9-week study, showed that informal meditation practice improved an individual’s positive emotions. Further, Hunt et al. [61] conducted a meta-analysis showing that meditation had a positive relationship with positive affect and that the effect size ranged from medium to strong. Zeng et al. [62] also showed that meditative practices were associated with the activation of the left prefrontal cortex, which, in turn, improved positive affect. Hence, it appears to be consensual that meditation brings benefits for individuals’ affective states as it appears to be a significant predictor of positive affect. However, the impact of meditation is not limited to positive affect. Diverse studies have shown that it also improves mood, happiness, life satisfaction, and health [21].

### 2.3. The Relationship Between Meditation and Health

In line to disentangle the role of positive affective states on the relationship between meditation practices and health, we focused on two health indicators, namely vitality and mental health, as each one appears to be relevant for individuals’ functioning [63].

Vitality is an indicator of physical health and includes aspects of physical functioning [63]. At work, vitality is also an indicator of the absence of role limitations due to physical health problems, and the absence of bodily pain that enables the individual’s social and role functioning [64]. Furthermore, mental health is related to the individual’s social functioning and includes the absence of role limitations due to emotional problems, and well-being [65].

Fredrickson et al. [17] suggested that meditation positively affects physical and mental health. Workers who practice meditation derive energy from it, and their vitality increases. Based on the approach of Fredrickson et al. [15,17], meditation counteracts negative affect and enhances an individual’s ability to cope with stressful situations and better regulate their affective responses. Indeed, meditation has been shown to decrease the experience of negative physical and mental health states (e.g., depression, anxiety, or stress; [66]). Hence, we argue that meditation practices during micro-breaks trigger positive affect and this, in turn, promotes employees’ physical and mental health.

### 2.4. The Relationship Between Positive Affect and Health

The relationship between positive affect and health is supported by the broaden-and-build theory [18]. Accordingly, positive emotions broaden the individuals’ cognitive and behavioral spectrum, influencing positive behaviors and attitudes. That is, positive emotions expand a set of thoughts, dispositions, motivations, and actions that arise in the mind spontaneously [18]. Hence, those who experience positive emotions momentarily enjoy an expansion of their human capital and feel more energetic (i.e., vitality) and mentally healthy [67]. For example, a happy individual is more likely to feel vigorous at work than someone anxious [68]. Additionally, someone who feels positive affect more regularly will tend to feel psychologically healthier, less distressed, or more anxious [21,69]. With more positive emotions, individuals achieve a greater and broader degree of perception, including more inclusive and connected social perceptions, and tend to feel more relaxed and engage in expansive behaviors that contribute to both physical and mental functioning [70].

Moreover, Fredrickson [17] argues that positive emotions develop personal resources contributing to the so-called upward spirals of positive emotions, as these can positively influence well-being and other positive long-term outcomes, such as mental and physical health. The emergence of positive results can occur through the continuity and increase of positive emotions, where the upward spirals lead not only to the emergence of even more positive emotions but also to their buffering effect on the relationship between negative emotions and negative outcomes [15]. For example, Junça-Silva and Caetano [71] demonstrated that positive affect arouses upward spirals toward well-being and, on the other hand, negative affect leads to a downward spiral of depression and anxiety. They highlighted that positive emotions are not only a good feeling but also increase well-being and expand available resources for employees to feel better and more energized (e.g., vitality). Hence, the experience of positive emotions, such as enthusiasm or joy, allows employees not only to feel good but also to be able to protect and develop resources that can contribute to their vitality and mental health (e.g., [72]).

Furthermore, Hobfoll et al. [73] argued that positive affect is crucial as it promotes successful outcomes in various life domains, including health. Indeed, positive affect facilitates approach behavior that, in turn, enhances vitality and employees’ mental health (e.g., [74]). Concerning this, some scholars have argued that positive affect improves health because it is a motivational force that energizes employees (i.e., vitality) and decreases impaired psychological states (e.g., depression; [75]).

### 2.5. The Relationship Between Meditation and Health: Indirect Effect via Positive Affect

Meditation practice triggers positive affect and has beneficial effects on employees’ health [6]. Moreover, positive affect has a relationship with employee behaviors (momentary or long-term behaviors), based on positive stimuli (such as meditation) that significantly influence their health [76].

We argue that meditation practices during worktime may be performed in informal micro-breaks—short breaks from work [77]—that serve to enhance individuals’ affective states (i.e., positive affect) and in turn their physical and mental health. As described before, meditation enhances the individual’s sustained focus of attention, and this can enable the emergence of contents previously inaccessible to awareness [35], initiating a process of accessibility to unknown states of consciousness, which culminates simultaneously with the access to positive affective levels (e.g., [59]).

Further, meditation develops positive psychological characteristics as it increases the individual’s ability to reduce distracted and absorbed thoughts, such as daydreaming [78], enhances self-awareness [52] which influences positive affect [6], and, as a result, energizes individuals’ and contributes to their mental health (e.g., [22]). Meditation, therefore, is associated with increased health (vitality and mental health) due to the higher levels of positive affective experiences. Based on the arguments, we expect that:

**Hypothesis 1:**  *Meditation will be indirectly related to the employee’s (a) vitality and (b) mental health via positive affect*.

### 2.6. The Moderating Role of Sleep Quality

Recent research has emphasized the need to clarify the relationship between meditation and its subsequent affective and health-related outcomes (e.g., [26,76]) by examining conditions that may amplify or buffer its effects [21]. One such condition is sleep quality, which plays a critical role in how individuals appraise and respond to work-related experiences (e.g., [79]).

Sleep is a dynamic recovery process [24] that encompasses both objective and subjective dimensions. It includes quantitative aspects, such as sleep duration, latency, and the number of awakenings, as well as qualitative aspects, such as sleep depth and perceived restfulness [80]. Scholars commonly distinguish between two dimensions of sleep: quality and quantity [81]. Sleep quality refers to an individual’s subjective evaluation of their sleep experience, whereas sleep quantity pertains to the total hours of sleep obtained [82].

We propose that sleep quality moderates the effects of meditation on positive affect. As discussed earlier, meditation enhances awareness and attentional control, enabling individuals to reappraise stimuli in ways that promote positive affect (e.g., [15]). However, this effect may be particularly pronounced when individuals have experienced poor sleep. On days following inadequate sleep, individuals may rely more heavily on meditation to regulate their affective state, making positive affect more dependent on meditation practice.

The effort–recovery model [83] suggests that individuals must recover resources depleted by daily work demands. Meditation provides an opportunity for such recovery by fostering self-regulatory processes that facilitate detachment from work-related stressors [26]. Meijman et al. [84] identified two key mechanisms through which meditation enhances self-regulation: (1) decoupling of the self from experiences and (2) interoceptive awareness.

Decoupling of the self from experiences refers to a shift in perspective that allows individuals to observe their thoughts and emotions without becoming entangled in them [27]. Meditation promotes this “reperceiving” process by cultivating present-moment awareness and nonjudgmental observation of both external events and internal states [34]. This meta-awareness enables individuals to acknowledge their thoughts and emotions objectively, minimizing extreme emotional reactions. For instance, meditation may help individuals reappraise a poor night’s sleep in a neutral, accepting manner, reducing concerns and negative attitudes associated with insufficient rest [26]. By fostering a nonjudgmental perspective, meditation may mitigate the negative psychological effects of poor sleep and facilitate psychological detachment from work-related stressors. Thus, meditation may enhance individuals’ ability to accept and reframe their sleep experience, leading to a stronger association between meditation and positive affect on days of poor sleep compared to days of good sleep.

Additionally, meditation enhances interoceptive awareness—an individual’s sensitivity to internal physiological states, including respiration, circulation, and proprioception [84]. This heightened awareness plays a crucial role in maintaining homeostasis and regulating emotions, behaviors, and cognitive processes [85]. Individuals who are more attuned to early signs of stress or fatigue are better equipped to engage in recovery-enhancing behaviors [26].

Building on these arguments, we propose that meditation fosters awareness of internal cues signaling poor sleep while simultaneously promoting a nonjudgmental attitude toward sleep quality. This dual process may facilitate resource acquisition, shaping more adaptive affective responses. Accordingly, we hypothesize that:

**Hypothesis 2:**  *Sleep quality will moderate the relationship between meditation and positive affect. Specifically, the relationship between meditation and positive affect will be stronger when sleep quality is poor compared to when sleep quality is good*.

### 2.7. The Moderated Mediation Hypothesis

Meditation may enhance psychological functioning on days following poor sleep quality by promoting awareness that influences positive affective responses [57]. The benefits of meditation are likely to be amplified when it facilitates awareness of internal states, such as fatigue, enabling individuals to regulate their emotional reactions and, in turn, improving their health outcomes [11]. Moreover, sensitivity to internal stimuli, such as respiratory patterns or fatigue, plays a key role in maintaining homeostasis [86] and promoting effective self-regulation, both of which are essential for optimizing psychological functioning and health [85]. Consequently, interoceptive awareness can serve as an indicator of insufficient recovery, signaling the need to engage in meditation to facilitate affective regulation and reduce the risk of health impairments.

Building on these theoretical considerations, we hypothesize that the indirect effect of daily meditation on health (mediated by positive affect) will be more pronounced on days following poor sleep quality, compared to days with adequate rest.

**Figure 1:**
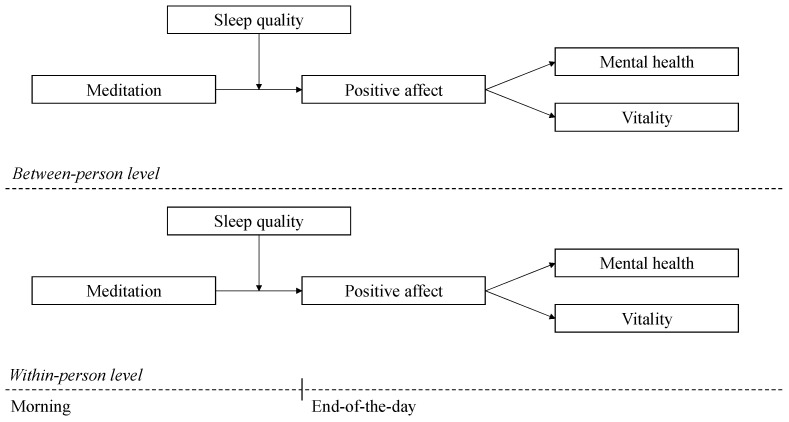
The hypothesized moderated mediation model.

**Hypothesis 3:**  *Sleep quality will moderate the indirect effect of meditation on employee (a) vitality and (b) mental health (via positive affect). Specifically, the indirect relationship of meditation with (a) vitality and (b) mental health via positive affect will be stronger when sleep quality is poor compared to when sleep quality is good—where the highest level of positive affect would be experienced with a higher level of meditation and a poorer quality of sleep (Figure 1)*.

## 3. Method

### 3.1. Procedure and Participants

Data were collected using a snowball sampling approach from regular meditators employed in public healthcare services. The data collection process was conducted in two phases. In the first phase, participants completed a general survey assessing socio-demographic characteristics (e.g., age, gender, working hours, and tenure) and their daily meditation practices. One week later, in the second phase, participants were invited to complete surveys twice daily over a five-day workweek (Monday to Friday).

During this phase, a morning survey was distributed at 11 a.m., in which participants reported their meditation practice (mindfulness meditation), duration, and perceived control over it. A second survey was sent at 9 p.m., prompting participants to report their affective experiences throughout the workday as well as their levels of vitality and mental health. Given the variability of work shifts in healthcare settings, participants were allowed to respond to the morning survey until 2 p.m. and the evening survey until 11 p.m. Responses submitted outside these time frames were not accepted. To maximize participation, two reminder emails were sent each day. The time lag between the morning and evening surveys helped establish temporal separation between the predictor variable (meditation) and the outcome variables (affect and health; [87]), thereby mitigating common method bias [88,89].

The study invitation, accompanied by an information letter emphasizing voluntary participation and confidentiality, was initially distributed to 11 nurses from a public hospital. These were asked to forward the invitation to their colleagues (nurses and doctors who practiced meditation). Through snowball sampling, an additional 49 individuals volunteered, resulting in an initial pool of 60 participants. Nine individuals were excluded as they did not practice meditation at work. Of the remaining 51 eligible participants, seven were excluded due to incomplete survey responses, resulting in a final sample of 44 healthcare professionals (response rate: 73.3%).

The sample size was calculated using GPower statistical power analysis software (GPower 3.1.9.7; Kiel University, Kiel, Germany [90]) for a linear multiple regression model with three predictors (i.e., the three socialization tactics). The input parameters were as follows: statistical test = *t*-tests: linear multiple regression; effect size f^2^ = 0.15; α error probability = 0.05; power (1 − β error probability) = 0.80; and number of predictors = 2. Based on these parameters, the required sample size was determined to be 43 participants. Therefore, the sample of 44 was considered sufficient for testing the model.

The final sample consisted of 44 healthcare workers, comprising 73% nurses and 27% doctors. Participants completed a total of 220 daily surveys, with each morning survey matched to its corresponding end-of-day survey. The majority of participants were female (72.7%), with a mean age of 45.87 years (SD = 8.21) and an average job tenure of 15.83 years (SD = 14.31). On average, they worked 38.48 h per week (SD = 10.21).

### 3.2. Measures

All English measures were translated into Portuguese using a double-blind back-translation procedure [91]. First, two language experts who were fluent in both Portuguese and English translated the surveys, and then two researchers reviewed them to ensure the appropriate content and face validity of the surveys. All measures were scored on a five-point Likert scale.

### 3.3. Daily Meditation

Participants were asked to assess their morning meditation, using a three-item survey measuring the degree to which their thoughts were focused on the meditation practice [1]. Participants rated the degree to which they agreed with the following items about their experience while meditating: “This morning while meditating, I was focused on my breathing”, using a five-point scale (1 = very slightly or not at all, 5 = extremely). The average coefficient alpha across days was 0.76. Moreover, they were subsequently asked to report the number of minutes they had meditated. Responses to average daily practice varied and ranged from 5 min to 60 min (M = 39.93, SD = 16.63).

### 3.4. Positive Affect

We measured positive affect at the end of the day with the 8-item Multi-Affect Indicator [92] (e.g., enthusiasm). Responses were given concerning the frequency of positive affect experienced on that day at work, using a five-point Likert scale that ranged between (1) never and (5) always. The average coefficient alpha across days was 0.93.

### 3.5. Vitality and Mental Health

We used the SF-36 health survey [93] to assess vitality (four items) and mental health (five items) at the end of the day. A sample item for vitality is “How much of the time during today did you have a lot of energy?”, and for mental health is “How much of the time during today, have you been happy?”. Participants had to respond using a five-point Likert scale, ranging from none of the time (1) to all of the time (5). The average coefficient alpha across days for the vitality dimension was 0.92, and for mental health was 0.89.

### 3.6. Sleep Quality

We measured sleep quality in the morning using a shortened version of the Pittsburgh Sleep Quality Index (PSQI) [80]. We adapted the items for night-specific assessment by referring to the previous night. For each person and each night, we calculated a day-specific sleep quality score. In line with Jahrami et al. [80] (p. 194), we used two items, answered on a five-point Likert scale, to assess the participants’ subjective component of sleep: sleep quality [“How would you rate the quality of your previous night’s sleep?”; 1 (very bad) to 5 (very good)] and restfulness [“This morning, how much of a problem has it been for you to keep up enough enthusiasm to get things done?”; 1 (a very big problem) to 5 (not at all)]. This measure is widely used to assess sleep quality in organizational research [94,95]. Higher values indicated higher day-specific sleep quality. The average coefficient alpha across days was 0.88.

### 3.7. Control Variables

We controlled for gender and age because these variables are related to health and affect [21]. We also controlled for the day of data collection (from Monday to Friday) as it seems to influence the effectiveness of meditation results [96,97] and mental health [74].

### 3.8. Analytical Strategy

Given that our study design included days nested within individuals, we used multilevel analyses to test the proposed model. First, we calculated the analysis of variance components. We found significant variance in morning meditation (ICC = 0.29), daily positive affect (ICC = 0.39), daily vitality and mental health (ICCs = 0.66, 0.58), and night’s sleep quality (ICC = 0.63). This evidenced significant variation both at the within- and between-person levels. Thus, we proceeded with the multilevel analyses.

Our hypotheses were tested through the macro–Multilevel Mediation (MLMed), in SPSS, version 28 [98]. Moreover, we used the Monte Carlo resampling method [99] to assess the statistical significance of the indirect effect. This approach represents the asymmetric complexity of the sampling distribution of an indirect effect and demonstrates superior precision over the Sobel test [100].

## 4. Results

### 4.1. Multilevel Confirmatory Factor Analysis

Before investigating our moderated mediation model, we conducted several confirmatory factor analyses to evaluate the convergent and discriminant validity of night sleep quality, morning meditation, daily positive affect, and daily vitality and mental health. Table 1 shows that the hypothesized five-factor model (χ^2^ (160)) = 540.415, Tucker–Lewis index [TLI] = 0.99, comparative fit index [CFI] = 0.99, root mean square error of approximation [RMSEA] = 0.10, standardized root mean square residual [SRMR_within_] = 0.06 and [SRMR_between_] = 0.07) meet conventional standards for acceptable fit [101], and this fit was significantly better than the alternative models. To test differences in the model fit, we considered both the significance of the change in chi-square and differences of at least 0.01 on TLI or CFI or a change of at least 0.01 on RMSEA. As such, these findings demonstrated additional evidence for the validity of the measures.

### 4.2. Descriptive Statistics

The descriptive statistics and correlations are presented in Table 2.

### 4.3. Hypothesis Testing

As we mentioned before, to test the hypotheses, we considered the hierarchical structure of the data, in which daily data were nested within individuals.

### 4.4. Testing Within-Person Indirect Effects

Hypothesis 1 proposed that morning meditation would have an indirect effect on end-of-day (a) vitality and (b) mental health through positive affect. After controlling for positive affect, the direct effect of meditation on vitality became nonsignificant (γ = 0.07, SE = 0.06, *p* = 0.21), indicating that 78% of the variance in vitality was accounted for by all day-level factors. To further examine the significance of this indirect effect, we applied a bootstrapping approach to estimate the indirect effects of meditation on vitality [99]. The bootstrapped unstandardized indirect effect of 0.96 through positive affect was significant (95% CI [0.19, 1.78]).

Similarly, the within-person effect of meditation on mental health became nonsignificant after controlling for positive affect (γ = −0.01, SE = 0.05, *p* = 0.94), suggesting that 73% of the variance in mental health was explained by all day-level factors. The bootstrapped indirect effect of meditation on mental health via positive affect was also significant (95% CI [0.14, 1.32]), with an unstandardized estimate of 0.72. These findings indicate that the beneficial effects of morning meditation on daily health indicators (vitality and mental health) were fully mediated by employees’ positive affect throughout the workday, providing empirical support for Hypotheses 1a and 1b.

### 4.5. Examining the Moderating Effect of Sleep Quality

**Figure 2:**
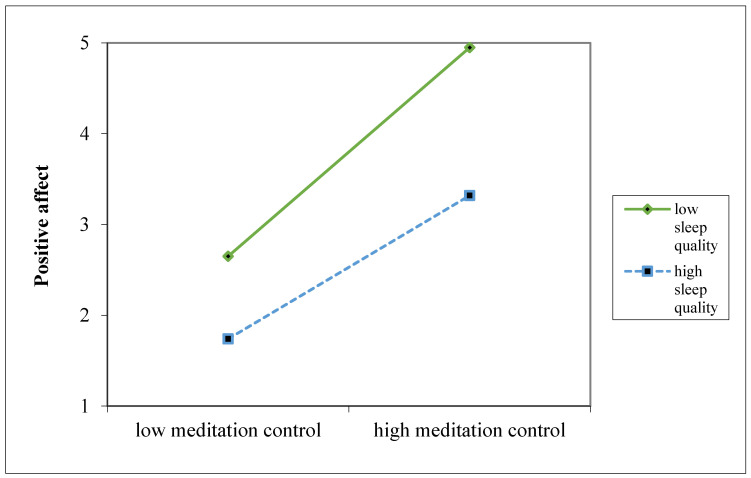
The moderating effect of sleep quality on the relationship between morning meditation and positive affect.

Hypothesis 2 posited that sleep quality from the previous night would moderate the relationship between morning meditation and positive affect. The results revealed a significant within-person interaction effect on positive affect (γ = −0.36, SE = 0.16, *p* = 0.02) (see Table 3). Furthermore, simple slope analyses [102] indicated that the association between morning meditation and positive affect was stronger when sleep quality was poor (*t* = 25.71, *p* < 0.001) compared to when sleep quality was good (*t* = 7.21, *p* < 0.001) (see Figure 2). These findings provide empirical support for Hypothesis 2, suggesting that morning meditation is particularly beneficial in enhancing positive affect when individuals experience inadequate sleep.

To test the moderated mediation effects proposed in Hypotheses 3a and 3b, we conducted a Monte Carlo analysis with 20,000 simulations. The results confirmed that sleep quality significantly moderated the indirect effect, supporting a moderated mediation model for both vitality (γ_within_ = −0.31, 95% bias-corrected bootstrap CI [−0.57, −0.05]) and mental health (γ_within_ = −0.23, 95% bias-corrected bootstrap CI [−0.43, −0.04]). Thus, Hypotheses 3a and 3b were supported.

Additionally, we applied Rockwood et al.’s [99] approach to estimate the conditional indirect effect at high (+1 standard deviation) and low (−1 standard deviation) levels of sleep quality. The results indicated that the indirect effect of morning meditation on vitality via positive affect remained significant at both high and low levels of sleep quality (estimate = 0.30, SE = 0.08, *p* < 0.001; estimate = 0.27, SE = 0.07, *p* < 0.001). Moreover, bootstrapping results confirmed that the indirect effect through positive affect was significant at both levels of sleep quality, as the 95% confidence intervals did not include zero [0.16, 0.49; 0.15, 0.41] (see Figure 2).

**Figure 3:**
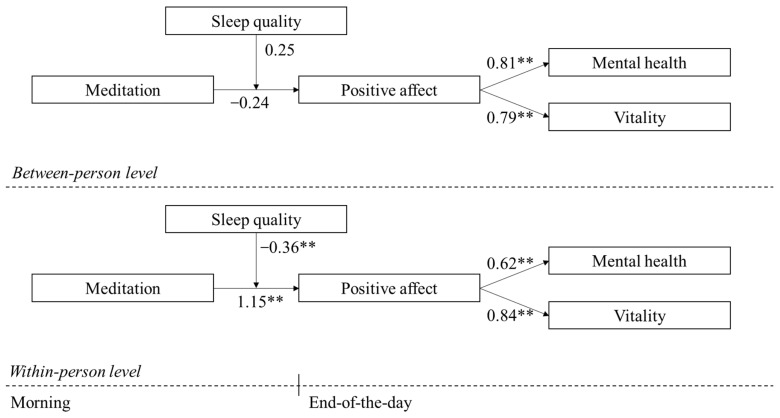
Multilevel moderated mediation results. (** *p* < 0.001).

Moreover, the indirect effect of morning meditation on end-of-day mental health via positive affect remained significant at both high and low levels of sleep quality (estimate = 0.31, SE = 0.08, *p* < 0.001; estimate = 0.27, SE = 0.07, *p* < 0.001). Bootstrapping results further confirmed the significance of these indirect effects, as the 95% confidence intervals did not include zero [0.17, 0.48; 0.15, 0.41]. Therefore, Hypotheses 3a and 3b were supported (see Figure 3).

## 5. Discussion

This study investigates the role of morning meditation during micro-breaks in relation to affective and health-related outcomes. Specifically, it examines positive affect as a mediating mechanism linking morning meditation to health-related indicators (i.e., vitality and mental health) and explores the moderating effect of sleep quality on these relationships.

Overall, the findings support the proposed moderated mediation model. Morning meditation during micro-breaks is indirectly associated with improved health-related outcomes through its impact on positive affect. Moreover, the direct effect of morning meditation on positive affect is moderated by sleep quality, such that employees who engage in morning meditation experience greater positive affect, particularly following a night of poorer sleep compared to a well-rested night.

### 5.1. Theoretical Contributions

Overall, the findings contribute to a deeper understanding of the role that meditation practices during the workday may play in influencing affective and health-related outcomes [103]. The results emphasize that meditation can be a valuable tool for employees [104], offering both immediate benefits, such as improved positive affect at work, and longer-term effects, including enhanced vitality and mental health. These findings extend the current literature on the benefits of meditation (e.g., [32,105]) by linking meditation to affective and health-related outcomes and providing empirical evidence that positive affect is a key mechanism through which meditation influences health. Previous research has demonstrated that meditation enhances awareness and attention to present cues, which can facilitate positive affective reactions and influence subsequent behaviors (e.g., [11,76,106]). In alignment with the growing body of research on workplace meditation (e.g., [21]), our findings establish daily meditation as a promising practice within the occupational healthcare domain, offering potential benefits for both employees and organizations [105].

This study extends prior research on the relationship between meditation and health in three significant ways. First, the results show that morning meditation, practiced during micro-breaks from work, can help employees restore their affective resources, leading them to feel more energized and mentally balanced by the end of the workday. Micro-breaks have been found to positively influence employees’ affective outcomes, such as emotional state [107] and work engagement [108], helping to reduce fatigue and increase vigor [109]. Consistent with this evidence, micro-breaks incorporating meditation may offer similar benefits. Meditation enables individuals to achieve positive changes in their affective states by enhancing awareness and focus on the present moment [103]. This heightened awareness enables individuals to anticipate negative events while savoring positive experiences [104], thereby contributing to their overall physical and mental health [106].

The modal model of emotion [55] supports these findings by emphasizing the role of stimuli (i.e., meditation), attention, and appraisal in emotion regulation. Empirical studies (e.g., [76]) have shown that meditation promotes mental and muscular relaxation, allowing individuals to self-regulate through focused attention [32]. Meditation has been associated with improved emotional regulation, positive affect, mood, happiness, and psychological well-being [1,17]. Meditation practices appear to influence brain regions associated with positive affect, and daily meditation during work breaks can help foster positive affect [105].

Second, our findings establish positive affect as a mediating mechanism that explains how meditation is related to health outcomes. Daily meditation improves employees’ emotional well-being at work [103], helping them regulate their emotions [104], which, in turn, enhances their vitality and mental health. Consistent with Fredrickson’s broaden-and-build theory [18], positive affect fosters vigor (e.g., vitality) by broadening cognitive and behavioral repertoires. This expanded range of responses encourages individuals to engage in positive, resource-acquiring behaviors, essential for maintaining well-being. As employees experience increased positive affect, they become more energized and capable of sustaining balanced mental health [15]. The indirect effect of meditation on health through positive affect emphasizes the potential benefits of incorporating meditation practices during work breaks, not only for improving employees’ emotional states but also for enhancing their overall health.

Third, we expand the understanding of meditation’s role in the workplace by examining how its interaction with sleep quality influences affective and health outcomes. Sleep quality is crucial and has been shown to influence how employees feel during the workday [109,110,111]. In demanding fields, such as healthcare, sleep quality is especially important [110,111,112]. Given the increasing demands of modern work life [109,113], understanding how sleep quality interacts with meditation is increasingly critical.

The results show that morning meditation is more strongly associated with positive affect and health outcomes when sleep quality is poor. Specifically, meditation interacts with sleep quality such that positive affect increases when individuals engage in more meditation following a poor night’s sleep, compared to a good night’s sleep. This interaction underscores the importance of meditation as a tool for improving well-being when sleep quality—an essential component of recovery [80,114]—is inadequate. Meditation promotes present-moment awareness and nonjudgmental attitudes [85], allowing individuals to derive greater resources from their experiences [27]. Additionally, meditation enhances interoceptive awareness, helping individuals focus on internal stimuli (e.g., fatigue from poor sleep; [115]), and improves self-regulation to maintain internal homeostasis—critical for health [116]. Thus, even when sleep quality is poor, morning meditation enables employees to experience positive affect and feel healthier.

This study provides empirical evidence supporting the role of meditation as an effective workplace intervention for enhancing positive affect and health outcomes. By identifying sleep quality as a key boundary condition, we contribute to a more nuanced understanding of when meditation is most beneficial. Our findings have significant implications for practice, particularly in high-stress environments such as healthcare, where optimizing employee well-being is crucial for both individual and organizational performance.

### 5.2. Practical Contributions

This study explores the role of meditation in enhancing health through positive affect in healthcare environments characterized by high daily job demands. The findings underscore the importance of integrating both meditation and sleep quality interventions into occupational health strategies to improve healthcare workers’ well-being. Given the critical role of sleep in cognitive functioning and emotional regulation, healthcare managers should implement targeted initiatives to raise awareness about its significance. For instance, organizations could foster a culture of sleep awareness through structured workshops, training sessions, seminars, and strategic communication campaigns—leveraging social media and workplace displays to reinforce key messages. In addition, healthcare institutions could implement structured meditation programs during shift breaks, integrate sleep hygiene training into wellness initiatives, and cultivate a workplace culture that prioritizes both mindfulness and adequate rest. These efforts would be particularly beneficial for employees struggling with insomnia, shift workers (e.g., nurses and doctors), and individuals with poor sleep hygiene, such as those who engage in late-night screen exposure.

Moreover, promoting meditation as a coping mechanism for managing daily work-related stress can further enhance well-being. Meditation has been shown to facilitate emotional regulation and foster positive affect [58], which, in turn, contributes to improved health outcomes. Encouraging meditation practices could be especially valuable for individuals facing unique challenges, such as new mothers experiencing sleep deprivation in the early postpartum period. By integrating these interventions, healthcare organizations can create a healthier workforce, ultimately benefiting both employees and the quality of patient care.

### 5.3. Limitations and Future Directions

One limitation of this study is the use of snowball sampling, which may have introduced selection bias. Future research should consider alternative sampling methods to enhance representativeness. Additionally, the reliance on self-report measures for variables such as sleep quality and affective well-being may have led to response biases and introduced the potential for common method bias. However, several strategies were implemented to mitigate this bias, such as conducting multilevel factorial analyses. Integrating objective measures, such as wearable trackers, would improve data robustness by providing more precise and reliable assessments of these constructs. Future studies should explore the use of multimethod approaches to strengthen the validity of findings. Additionally, despite the use of a daily diary design with two daily data collections, data on the mediator and outcomes were collected simultaneously at the end of the day, which may introduce temporal bias. Future studies should aim to collect data at different time points, particularly for the mediator and outcome variables, to address this potential bias.

Future research should prioritize longitudinal studies to examine the sustained effects of meditation over time. While our study provides valuable insights into the immediate benefits of morning meditation on daily well-being, a longitudinal approach would allow for a more comprehensive understanding of how these effects evolve and whether they lead to lasting improvements in vitality, mental health, and resilience. Investigating the long-term impact of meditation could also help identify potential cumulative benefits and clarify the mechanisms underlying its effectiveness in workplace settings.

Moreover, future studies should explore the productivity benefits of meditation, including performance indicators such as adaptive performance. A comparative approach could also be valuable, analyzing employee performance on days with meditation practices versus days without. Lastly, future studies should investigate which specific meditation techniques are most beneficial in organizational contexts, as different techniques are associated with distinct outcomes and can help develop specific skills.

## 6. Conclusions

Drawing on multilevel data from healthcare workers, this study enhances the understanding of how morning meditation practices influence health outcomes, as well as the affective mechanisms and contextual conditions shaping this relationship. Overall, the findings support our initial hypotheses. Specifically, the results underscore morning meditation as a valuable micro-break that predicts end-of-day vitality and mental health. Moreover, they suggest that on nights with poorer sleep, engaging in meditation can serve as an effective strategy for enhancing positive affective states, ultimately contributing to greater subjective vitality and perceived mental health.

## Figures and Tables

**Table 1:** Comparison of measurement models.

Model		χ^2^ (*df*)	RMSEA	CFI	TLI	SRMR_within_	SRMR_between_	Comparison	Δχ^2^	Δ*df*	*p*
M1	5 latent factors	540.415 (160)	0.10	0.99	0.99	0.06	0.07	*-*	*-*	*-*	-
M2	3 latent factors	562.883 (167)	0.11	0.97	0.97	0.07	0.08	M2-M1	22.00	7	<0.001
M3	2 latent factors	1191.054 (169)	0.17	0.97	0.97	0.10	0.10	M3-M1	650.63	9	<0.001
M4	1 latent factor	2180.615 (170)	0.24	0.98	0.98	0.14	0.14	M4-M1	1640.20	10	<0.001

Note. RMSEA: root mean square error of approximation; CFI: comparative fit index; TLI: Tucker–Lewis index; SRMR: standardized root mean square residual; Best-fitting model in italics. M1: meditation, positive affect, vitality, mental health, and sleep quality fit load onto five separate latent factors. M2: vitality and mental health were loaded onto one latent factor plus meditation and positive affect were loaded onto one separate latent factor, and sleep quality loaded onto another latent factor. M3: vitality and mental health were loaded onto one latent factor, plus meditation, positive affect, and sleep quality loaded onto another latent factor. M4: all the variables (meditation, positive affect, vitality mental health, and sleep quality) were loaded onto one single factor.

**Table 2:** Means, standard deviations, and between- and within-person level correlations.

Variables	M	SD	1	2	3	4	5	6
1. Meditation	3.29	0.42	-	0.25 **	0.24 **	0.16 *	0.17 *	0.00
2. Positive affect	4.10	0.71	0.40 **	-	0.64 **	0.62 ***	0.63 ***	−0.31 **
3. Vitality	4.23	0.87	0.46 **	0.88 ***	-	0.88 ***	0.77 ***	−0.26 **
4. Mental health	4.47	0.73	0.41 **	0.85 ***	0.88 ***	-	0.69 ***	−0.23 **
5. Sleep quality	4.24	0.90	0.11	−0.10	−0.07	−0.18 *	-	−0.21 **
6. Time	-	-	−0.13	−0.32 **	−0.26 **	−0.22 **	−0.10	-
7. Age	45.87	8.21	−0.17	−0.05	−0.08	−0.07	−0.12	−0.17 *
8. Gender	-	-	0.16 *	0.39 **	0.42 **	0.35 **	−0.04	0.08

Note. Correlations below the diagonal are at the between-person level. Correlations above the diagonal are at the within-person level. Gener: 1-female; 2-male. *** *p* < 0.001, ** *p* < 0.01, * *p* < 0.05.

**Table 3:** Parameter estimates for the multilevel moderated mediation models.

	Model 1 Mediator (PA)	Model 1 Dependent (Vitality)	Model 2 Mediator (PA)	Model 2 Dependent (MH)
Within-person effects				
Mean Intercept	6.64 ***	−0.20	6.65 ***	0.54
Meditation	1.15 **	0.07	1.15 **	−0.01
Positive affect	-	0.84 ***	-	0.62 ***
Sleep quality	-	-	-	-
Meditation * sleep quality	−0.36 **	-	−0.36 **	-
Time	−0.00	0.01	−0.00	0.00
Gender	-	-	-	-
Age	-	-	-	-
Between-person effects				
Meditation	−0.24	0.23 ***	−0.24	0.08
Positive affect	-	0.79 ***	-	0.81 ***
Sleep quality	−1.27 **	-	−1.27 **	-
Meditation * sleep quality	0.26	-	0.25	-
Time	−0.24 ***	−0.00	−0.24 ***	0.04
Gender	0.41 *	0.20 *	0.41 *	0.01
Age	0.05	0.06	0.00	0.05
Variance of random components				
Random intercept	0.15 **	0.00	0.15 **	0.04 **
Residual variance	0.21 ***	0.16 ***	0.21 ***	0.10 ***
Direct effect				
Within-level	0.07 CI 95% [−0.04, 0.18]		−0.01 CI 95% [−0.09, 0.09]	
Between-level	0.23 *** CI 95% [0.10, 0.35]		0.08 CI 95% [−0.08, 0.24]	
Indirect effect				
Within-level	0.96 ** CI 95% [0.19, 1.78]		0.72 ** CI 95% [0.14, 1.32]	
Between-level	−0.19 CI 95% [−0.98, 0.58]		−0.19 CI 95% [−1.01, 0.60]	
Conditional indirect effect				
Within-level	−0.31 ** CI 95% [−0.57, −0.05]		−0.23 ** CI 95% [−0.43, −0.04]	
Between-level	0.20 CI 95% [−0.04, 0.46]		0.21 CI 95% [−0.04, 0.47]	
Model fit statistics				
AIC	533.11		477.79	
BIC	548.64		493.32	
−2LL	525.11		469.79	
Sample size	L_1_ = 440; L_2_ = 44			

Note. Maximum likelihood estimation with robust standard errors (MLR) was used in estimation. L_1_ = level 1, L_2_ = level 2. *** *p* < 0.001, ** *p* < 0.01, * *p* < 0.05. PA = positive affect; MH = mental health.

## Data Availability

The data presented in this study are available on request from the corresponding author. The data are not publicly available due to privacy or ethical restrictions.
